# The Johnstown Calamity

**Published:** 1889-06

**Authors:** W. F. Fundenburg, J. G. Templeton, H. W. Arthur, J. S. Goshorn, Lee S. Smith

**Affiliations:** 52 Sixth St., Pittsburgh, Pa.; 52 Sixth St., Pittsburgh, Pa.; 52 Sixth St., Pittsburgh, Pa.; 52 Sixth St., Pittsburgh, Pa.; Treasurer. 52 Sixth St., Pittsburgh, Pa.


### THE JOHNSTOWN CALAMITY.

PITTSBURGH, June, 1889.

*To the Dental Profession and Manufacturers and Dealers in Dental Goods:*

A terrible calamity has swept a once populous and prosperous city almost out of existence. Johnstown, Cambria Co., Pa., which, with its suburbs, contained about 25,000 inhabitants is in ruins, thousands of lives lost, and millions of dollars' worth of property destroyed.

In this ruin, our professional brethren have had their share. Johnstown contained ten practising Dentists; one lost his life, others lost parts of their families, most of them lost all their property, and all have lost their practice, at least for a long time to come.

The members of the profession in this vicinity, while recognizing the fact that nearly all have already contributed to the general relief fund, yet think that as a simple act of justice, the profession at large should step in to the relief of our professional brethren in distress. not as an act of charity, as that word is generally used, but in a higher sense, as brother to brother.

The undersigned have been appointed a Committee to present the matter to the profession and dental trade, and to receive subscriptions for purpose named.

We think the cause needs no urging on our part, believing that each and every one will be glad of the opportunity to cast in his mite.

We need hardly add that our action is taken without the knowledge of the sufferers at Johnstown.

Subscriptions may be sent to our Treasurer, and drafts, orders, etc., made payable to his order.

Very truly yours,

W. F. FUNDENBURG,
J. G. TEMPLETON,
H. W. ARTHUR,
J. S. GOSHORN,
LEE S. SMITH, Treasurer.
52 Sixth St., Pittsburgh, Pa.

